# Protection of Recombinant Mammalian Antibodies from Development-Dependent Proteolysis in Leaves of *Nicotiana benthamiana*

**DOI:** 10.1371/journal.pone.0070203

**Published:** 2013-07-23

**Authors:** Stéphanie Robert, Moustafa Khalf, Marie-Claire Goulet, Marc-André D’Aoust, Frank Sainsbury, Dominique Michaud

**Affiliations:** 1 Centre de recherche en horticulture, Département de phytologie, Université Laval, Québec City, Québec, Canada; 2 Medicago, Inc., Québec City, Québec, Canada; Kansas State University, United States of America

## Abstract

The expression of clinically useful proteins in plants has been bolstered by the development of high-yielding systems for transient protein expression using agroinfiltration. There is a need now to know more about how host plant development and metabolism influence the quantity and quality of recombinant proteins. Endogenous proteolysis is a key determinant of the stability and yield of recombinant proteins in plants. Here we characterised cysteine (C1A) and aspartate (A1) protease profiles in leaves of the widely used expression host *Nicotiana benthamiana*, in relation with the production of a murine IgG, C5-1, targeted to the cell secretory pathway. Agroinfiltration significantly altered the distribution of C1A and A1 proteases along the leaf age gradient, with a correlation between leaf age and the level of proteolysis in whole-cell and apoplast protein extracts. The co-expression of tomato cystatin *Sl*CYS8, an inhibitor of C1A proteases, alongside C5-1 increased antibody yield by nearly 40% after the usual 6-days incubation period, up to ∼3 mg per plant. No positive effect of *Sl*CYS8 was observed in oldest leaves, in line with an increased level of C1A protease activity and a very low expression rate of the inhibitor. By contrast, C5-1 yield was greater by an additional 40% following 8- to 10-days incubations in younger leaves, where high *Sl*CYS8 expression was maintained. These findings confirm that the co-expression of recombinant protease inhibitors is a promising strategy for increasing recombinant protein yields in plants, but that further opportunity exists to improve this approach by addressing the influence of leaf age and proteases of other classes.

## Introduction

Plant-based protein expression platforms are a useful answer to the growing demand for biological therapeutics and diagnostics worldwide [1], [2]. Plant cells, unlike bacteria or yeast, can correctly fold, assemble and modify complex proteins of mammalian origin, such as therapeutic and diagnostic antibodies. Plants also present advantages in terms of safety, capital investment, and ease of scaling-up compared to mammalian cell-based production systems [2], [3]. Much progress has been made to understand basic processes underlying heterologous protein expression in plants [4], [5], leading to tangible improvements in yield and product quality that have confirmed the position of plants as useful commercial expression hosts [6].

Originally most useful as a method to quickly screen transgene expression constructs [7], transient expression in agroinfiltrated plant leaves has developed into the fastest and most convenient production platform for plant-made biopharmaceuticals [8], [9]. This technique takes advantage of the ability of *Agrobacterium* species to transfer a transcriptionally competent segment of DNA, the T-DNA, into the plant host cell, where it is then directed to the nucleus [10]. A number of transient expression systems have been devised, that enable the production of milligram quantities of recombinant proteins within a few days using only a handful of plants [11]–[15]. These systems often make use of a wild relative of tobacco, *Nicotiana benthamiana*, as the expression host. Well known as a model for studies on plant-pathogen interactions [16], *N. benthamiana* grows quickly and is easily amenable to agroinfiltration for high-level protein production. As a result, this species has also become somewhat of a model plant for the transient expression of foreign proteins.

Despite major advances, the successful production of heterologous proteins in plants is sometimes limited by losses due to proteolysis [17]–[21]. Mammalian antibodies, in particular are known to be degraded *in planta* and during extraction, resulting in contamination of antibody preparations and, potentially, reduced fidelity of the protein product [22]–[25]. Proteases are involved in many aspects of plant biology, including development, nutrient remobilisation during senescence, and defense against pathogens [26]–[28]. As such, the impact of proteolysis on both the quantity and quality of recombinant proteins might vary with the developmental stage of plant tissues. Endogenous protease patterns in the host plant could also be influenced by the production method itself, which may significantly alter the leaf proteome and protease complement [29].

The co-expression of recombinant protease inhibitors has been proposed as a way to reduce the impact of plant proteases on recombinant protein yield [30]. Examples include protection of rhizosecreted antibodies in the growth medium of transgenic tobacco lines [31]; and protection during extraction from transgenic potato leaves [32], in which protein turnover was also modified to allow improved yield of transiently expressed recombinant proteins [33]. Most recently, this approach has been used to improve the expression of a transiently expressed antibody in *N. benthamiana* leaves through the co-secretion of protease inhibitors [34]. Little is still known, however about how this strategy relates to the host plant’s physiology and development, or to the reported substantial effects of agroinfiltration on protein expression and secretion [29]. Here we assessed the abundance and relative distribution of major protease activities in whole-leaf and apoplastic protein extracts of *N. benthamiana* through time and along the leaf age gradient, to investigate the influence of leaf age and agroinfiltration on recombinant protein quantity and quality. We used the human blood typing IgG antibody C5-1 as a model protein of clinical interest [15], [34], and two well characterized protease inhibitors from tomato, *Sl*CDI and *Sl*CYS8, to confirm the positive impact of co-expressed stabilising inhibitors *in planta*.

## Materials and Methods

### Expression Constructs and Agroinfiltration

**Figure 1:**
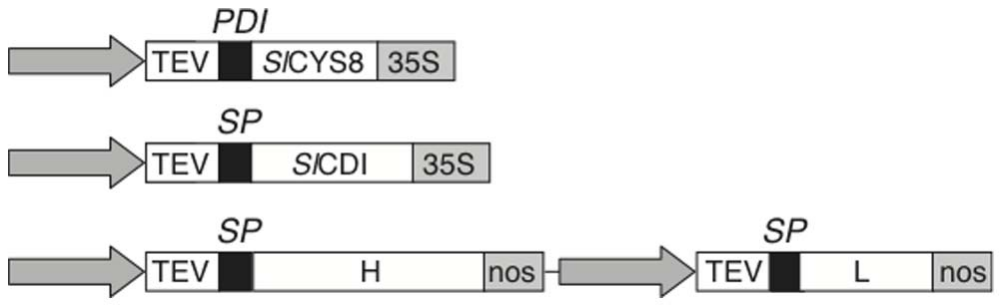
Binary vectors for C5-1, *Sl*CYS8 and *Sl*CDI expression. The constructs were devised for targeting the proteins to the apoplast. Each construct included a duplicated version of the CaMV 35S constitutive promoter (arrow), the TEV enhancer sequence (TEV), the respective protein-coding sequences (*Sl*CYS8, *Sl*CDI, H and L for C5-1 heavy and light chains) and a CaMV 35S terminator (35S) or nopaline synthase terminator (nos) sequence. Constructs for *Sl*CYS8 secretion included the coding sequence of alfalfa protein disulphide isomerase signal peptide (PDI). Constructs for C5-1 and *Sl*CDI included the native signal peptide (SP) of these proteins.

C5-1, *Sl*CDI and *Sl*CYS8 were expressed fused to an N-terminal signal peptide sequence allowing for cellular secretion. The coding sequences of *Sl*CDI and *Sl*CYS8 were placed downstream of a double *Cauliflower mosaic virus* (CaMV) 35S promoter, and flanked with the *Tobacco etch virus* (TEV) enhancer sequence and CaMV 35S terminator (Figure 1). The heavy and light chains of C5-1 were expressed using a similar gene cassette, except for the use of a nopaline synthase (nos) terminator and the insertion of both transgene sequences within a single binary vector (Figure 1). Vector constructs for *Sl*CDI and C5-1 including their native signal peptide were described earlier [29], [34]. For the cystatin, DNA sequences for *Sl*CYS8 [35] and the signal peptide of *Medicago sativa* protein disulphide isomerase (PDI) [36] were first PCR-amplified, and then fused by homology overlap extension PCR with the addition of 5′ BsrGI and 3′ NcoI restriction sites for insertion into a modified pUC plasmid such that the fusion was flanked in 5′ with CaMV 35S promoter and TEV enhancer sequences, and in 3′ with the CaMV 35S terminator sequence. The resulting expression cassette was transferred into the plant binary vector pCambia 2300 (CAMBIA, Canberra, Australia) as for *Sl*CDI and C5-1 chains.

The binary vectors were electroporated in *Agrobacterium tumefaciens* strain LBA4404, and the cultures maintained in the presence of 50 µg/ml kanamycin and 50 µg/ml rifampicin. For infiltration, cultures were grown to stable phase and collected by centrifugation at 2,000 *g*. Bacterial cells were resuspended in MMA (10 mM MES (2-[N-morpholino]ethanesulfonic acid), pH 5.6, 10 mM MgCl_2_, 100 µM acetosyringone) to an OD_600_ of 1.0 and incubated overnight at 4°C. The suspensions were let to stand at room temperature, and then pressure infiltrated into *N. benthamiana* leaves using a needleless syringe [37].

### Whole-leaf and Leaf Apoplast Proteins

Forty-two to 49 day-old greenhouse-grown plants were used for the experiments. Whole-leaf proteins were extracted in 400 µL of 10 mM MES buffer, pH 5.8, clarified by centrifugation at 10,000 *g* for 10 min at 4°C, and kept at –80°C until use. Total proteins were assayed according to Bradford [38], with bovine serum albumin as a protein standard (Sigma-Aldrich, Oakville ON, Canada). Leaf apoplast proteins were recovered as described [34], with some modifications. Freshly harvested leaves of the same age were weighed, washed in chilled double-distilled water, and submerged in chilled vacuum infiltration buffer (10 mM MES, pH 5.8). The leaves were then vacuum-infiltrated twice for 20 s with infiltration buffer, dried off to remove excess buffer, carefully rolled in a homemade swiss-roll cylinder, and centrifuged at 4°C for 10 min at 1,000 *g* to collect the vacuum infiltrate. Protein solutions were centrifuged at 6,000 *g* for 5 min at 4°C to discard *A. tumefaciens* cells. Protein content was assayed according to Bradford [38] with bovine serum albumin as a standard, and kept at –80°C until use.

### Protease Assays

Protease activities were assayed by the monitoring of substrate hydrolysis progress curves using the following synthetic fluorogenic substrates (Peptides International, Louisville KY, USA): *Z*-Phe–Arg–methylcoumarin (MCA) for C1A cathepsin L-like Cys proteinases, and MOCAc-Gly–Lys–Pro–Ile–Leu–Phe–Phe–Arg–Leu–Lys(Dnp)-d-Arg-NH_2_ for A1 cathepsin D/E-like Asp proteinases. Substrate hydrolysis reactions with whole-leaf and apoplast protein extracts (36 ng protein µL^−1^) were allowed to proceed at 25°C in 50 mM MES, pH 5.8, containing 10 mM l-Cys for Cys protease assays. Protease activity levels were monitored using a Fluostar Galaxy microplate fluorimeter (BMG, Offenburg, Germany), with excitation and emission filters of 360 and 450 nm, respectively, for the MCA substrate; or of 340 and 400 nm, respectively, for the MOCAc substrate. Three independent (biological) replicates were used for each assay.

### Immunodetections

*Sl*CDI, *Sl*CYS8 and C5-1 were immunodetected by Western blotting following 10% (w/v) SDS-PAGE and electrotransfer onto nitrocellulose membranes. Whole-leaf proteins were extracted in 50 mM Tris-HCl, pH 7.5, containing 150 mM NaCl and a commercial protease inhibitor cocktail (Roche, Laval QC, Canada) by disrupting tissue with tungsten carbide beads (QIAGEN, Mississauga ON, Canada) in a Mini-Beadbeater apparatus (BioSpec, Bartlesville OK, USA). *Sl*CDI was detected with commissioned polyclonal IgY antibodies raised in chicken against a hydrophilic surface loop of the protein [39] (Agrisera, Vännäs, Sweden) and alkaline phosphatase-conjugated rabbit anti-chicken IgY secondary antibodies (Sigma-Aldrich). *Sl*CYS8 was detected with commissioned polyclonal IgG antibodies raised in rabbit against bacterially expressed *Sl*CYS8 [35] (Agrisera) and alkaline phosphatase-conjugated goat anti-rabbit IgG as secondary antibodies (Sigma-Aldrich). C5-1 was detected with alkaline phosphatase-conjugated goat anti-mouse IgG (Sigma-Aldrich). Human IgG (Innovative Research, Burlington ON, Canada) were used as an antigen for C5-1 on activity blots, which was then detected with an alkaline phosphatase-conjugated anti-human IgG antibody (Sigma-Aldrich).

### C5-1 Quantification

Enzyme-linked immunosorbent assay (ELISA) plates for C5-1 quantification (Becton Dickinson, Mississauga ON, Canada) were coated with 3.75 µg/mL goat anti-mouse heavy chain-specific IgG1 (Sigma-Aldrich) in 50 mM carbonate buffer (pH 9.0) at 4°C for 16–18 h. The plates were washed three times in 10 mM phosphate-buffered saline containing 0.1% (v/v) Tween 20 (PBS-T), blocked through a 1-h incubation in 1% (w/v) casein in phosphate-buffered saline (PBS) (Pierce, Rockford IL, USA) at 37°C, and washed three times in PBS-T. A standard curve was generated for each plate with 0, 4, 8, 16, 24, 32, 40 and 60 ng/mL of purified mouse IgG1 (Sigma-Aldrich). All dilutions (controls and samples) were performed in a control extract from leaf tissue infiltrated with a mock inoculum so that any matrix effect was eliminated. The plates were incubated with protein samples and standard curve dilutions for 1 h at 37°C and then washed three times in PBS-T. The plates were then incubated with a peroxidase-conjugated goat anti-mouse IgG (H+L) antibody (0.2 µg/mL in blocking solution) (Jackson ImmunoResearch, West Grove PA, USA) for 1 h at 37°C. Washes with PBS-T were repeated, and the plates incubated with the 3,3′,5,5′-tetramethylbenzidine (TMB) Sure Blue peroxidase substrate (KPL; www.klp.org). The reaction was stopped by the addition of 1 N HCl, and the absorbance read at 450 nm. Each sample was assayed in triplicate, and antibody concentrations were interpolated in the linear portion of the standard curve. Three independent (biological) replicates were used for each assay.

### C5-1 Activity

ELISA plates for C5-1 activity monitoring (Immulon 2HB; ImmunoChemistry Technologies, Bloomington MN, USA) were coated with human IgG (Innovative Research) at a concentration of 2 µg/mL. The plates were washed in 0.85% (w/v) NaCl, blocked with 100 mM PBS containing 0.25% (w/v) casein and 0.01% (w/v) thiomersal at 37°C for 1 h, and washed three times in 0.85% (w/v) NaCl. All dilutions (controls and samples) were performed in a control extract obtained from leaf tissue infiltrated with a mock inoculum so that any matrix effect was eliminated. The plates were incubated with protein samples for 1 h at 37°C, washed three times in 0.85% (w/v) NaCl, and then incubated with peroxidase-conjugated goat anti-mouse IgG (H+L) antibodies (0.02 µg/mL in blocking solution) (Jackson ImmunoResearch) for 1 h at 37°C. Washes with 0.85% (w/v) NaCl were repeated, and the plates incubated with the TMB Sure Blue peroxidase substrate (KPL). The reaction was stopped by the addition of 1 N HCl, and the absorbance read at 450 nm. Each sample was assayed in triplicate. Three independent (biological) replicates were used for each assay.

### Statistical Analyses

Statistical analyses were performed using the SAS program, version 9.1 (SAS Institute, Cary NC, USA). Analysis of variance (ANOVA) and PROC MIXED procedures were used to compare protease activities. ANOVA and PROC GLM procedures were used to compare yields of C5-1 expressed alone or along with *Sl*CYS8 or *Sl*CDI. Contrast calculations were made when the ANOVA were significant at an alpha value threshold of 0.05.

## Results

**Figure 2:**
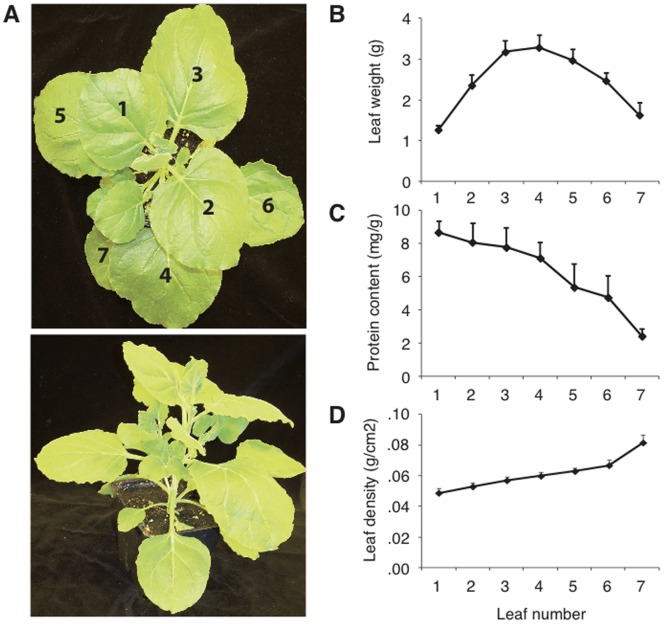
Characterisation of *N.* *benthamiana* leaf biomass. (A) Representative 49 days-old *N. benthamiana* plant (aerial and side views), with leaves numbered from the apex down. (B) Leaf weight given as a mean of eight measurements for each leaf position ± SE. (C) Mean protein content in total extracts from three biological replicates ± SE. (D) Leaf density calculated by dividing the weight of leaf discs by their area. Each point is the mean of three independent (biological replicate) values ± SE.

*N. benthamiana* as an expression host was first characterised in terms of leaf biomass for protein extraction, in order to gain insight into the relationship between physiological state along the leaf-age gradient and recombinant protein yield. The plants used were between 42 and 49 days-old, depending on the season, with eight fully expanded leaves arranged in an alternate pattern along the main stem. These leaves were numbered from the apex down so that leaf number correlates with leaf age (Figure 2A). The eighth leaf was not used as it was frequently senescent or damaged by contact with the pots of other plants. Measurement of physical characteristics revealed large variation in the biomass of each leaf (Figure 2B). There was a steady increase in leaf density with leaf age, but leaf mass was mostly determined by the leaf area. Protein content on a fresh weight tissue basis decreased significantly with leaf age (ANOVA, *P*<0.05), despite an increase in leaf density (Figure 2C and 2D).

### Endogenous Proteolytic Activities are Influenced by Leaf Age and Agroinfiltration

**Figure 3:**
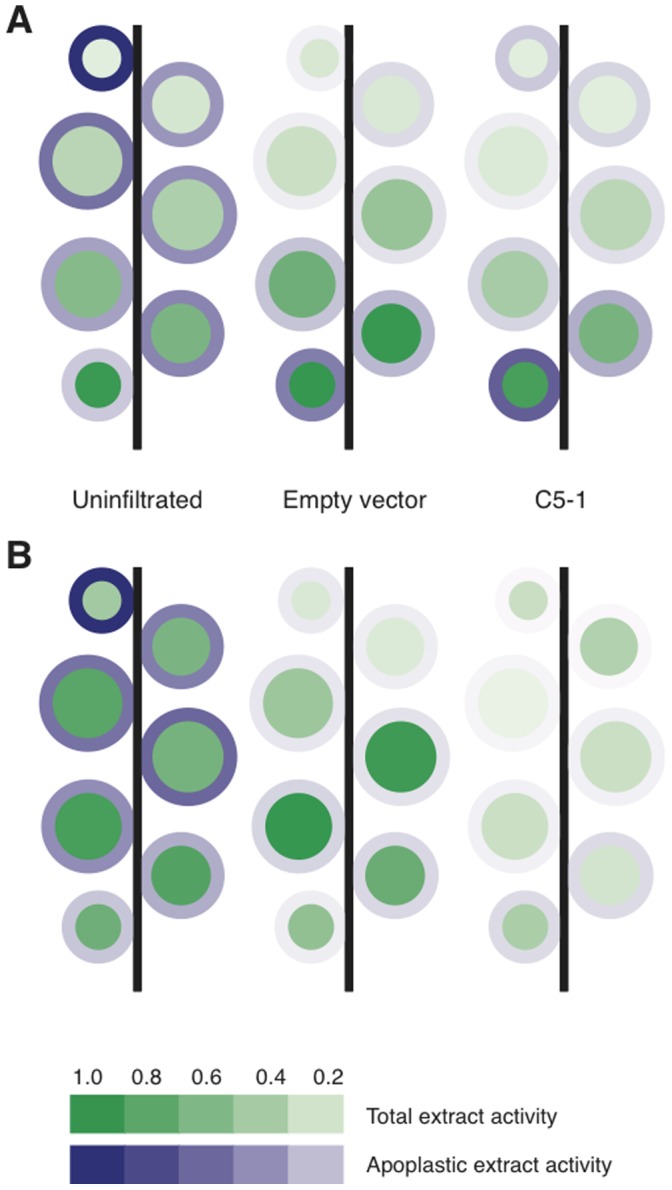
Major proteolytic activities in *N.* *benthamiana* leaves. Plants are represented as schematic diagrams where the area of each circle represents the weight of the corresponding leaf. Colour intensities indicate cathepsin L (A) and cathepsin D/E-like (B) activity levels detected for each leaf, relative to the maximum value obtained. Inner green circles represent whole-leaf extract activity, outer blue circles apoplastic extract activity. Each value is the mean of three independent activity values assayed using protease family-specific fluorogenic peptide substrates (fluorescence units/s.µg protein). Samples were taken at 6 dpi from plants infiltrated with Agrobacteria containing an empty vector, from plants infiltrated with Agrobacteria containing the C5-1 and P19 expression vectors, or from uninfiltrated plants of the same age.

Cathepsin D/E-like (A1 Asp protease) and cathepsin L-like (C1A Cys protease) activities have recently been shown to be the most abundant activities in total and apoplastic extracts of *N. benthaminana* leaves [34]. Here we assessed how these activities change with leave age, and whether they are affected by agroinfiltration or by expression of recombinant proteins (Figure 3). Since many glycoproteins of clinical interest are secreted to the apoplast, *in vitro* protease assays with cathepsin L and cathepsin D/E-specific fluorogenic peptides substrates were conducted for both whole-leaf and leaf apoplast protein extracts. A malate dehydrogenase assay first showed that less than 2% of the activity measured for this enzyme in whole-leaf extracts was found in the apoplast extracts (not shown), indicating a low level of contamination in apoplastic protein preparations as also observed previously [29]. As expected, cathepsin L and cathepsin D/E activities were detected in both apoplastic and total extracts (Figure 3). On a protein basis, cathepsin L activity was higher in apoplast extracts than in whole-leaf extracts of Leaf 1, at similar levels in Leaf 2 and Leaf 3, and two to thirteen times higher in whole-leaf extracts of older leaves, where extracellular activity decreased with leaf age (Table S1). By comparison, cathepsin D/E activity was nine to 120-fold higher in apoplast extracts. Considering that 80 to 100-fold more protein was found in whole-leaf extracts than in apoplast extracts, a major fraction of this proteolytic activity was, therefore, found in the apoplast. A significant leaf age-dependent increase in cathepsin L activity was observed for whole-leaf extracts (ANOVA, *P*<0.001), consistent with previous studies implicating C1A Cys proteases in physiological processes related to leaf maturation and senescence [27].

To investigate the effects of agroinfiltration and recombinant protein expression, leaves were infiltrated with either a bacterial culture containing an empty expression vector, or with a 1∶1 mixture of cultures harbouring expression vectors encoding either the C5-1 antibody or the P19 silencing suppressor (Figure 3). While there was no significant change compared to uninfiltrated plants in the profile of cathepsin L activity in whole-leaf extracts at six days post infiltration (dpi) (Figure 3A), cathepsin D/E activity significantly decreased in response to the control (empty vector) infiltration, and even further decreased following expression of C5-1 and P19 (ANOVA, *P*<0.001) (Figure 3B). A significant decrease of both cathepsin L and cathepsin D/E activities was observed in apoplast extracts of agroinfiltrated leaves (ANOVA, *P*<0.05), although cathepsin D/E activity now no longer decreased along the leaf age gradient and cathepsin L activity actually increased with leaf age. Together, these results suggest that major proteolytic activities are modified by both agroinfiltration and heterologous protein expression in *N. benthamiana* leaves. In particular, extracellular protease activity patterns along the leaf age gradient were extensively modified during the transient expression phase.

### The Antibody-stabilising Effect of Co-expressed Protease Inhibitors is Leaf Age-dependent

The co-expression of protease inhibitors has recently been shown to improve the accumulation of transiently expressed C5-1 in *N. benthamiana* leaves [34]. Here we monitored the impact of co-expressed inhibitors along the leaf age gradient to measure the potential of this approach at the plant scale, in relation with leaf development, biomass, and extractable protein yield. Two tomato inhibitors were used for the assays: the eighth domain of multicystatin *Sl*CYS8 to target cathepsin L proteases [35], and the Kunitz cathepsin D inhibitor *Sl*CDI to target cathepsin D/E proteases [40]. C5-1 was co-expressed with P19 and either inhibitors, after mixing Agrobacteria cultures containing the corresponding expression vectors.

**Figure 4:**
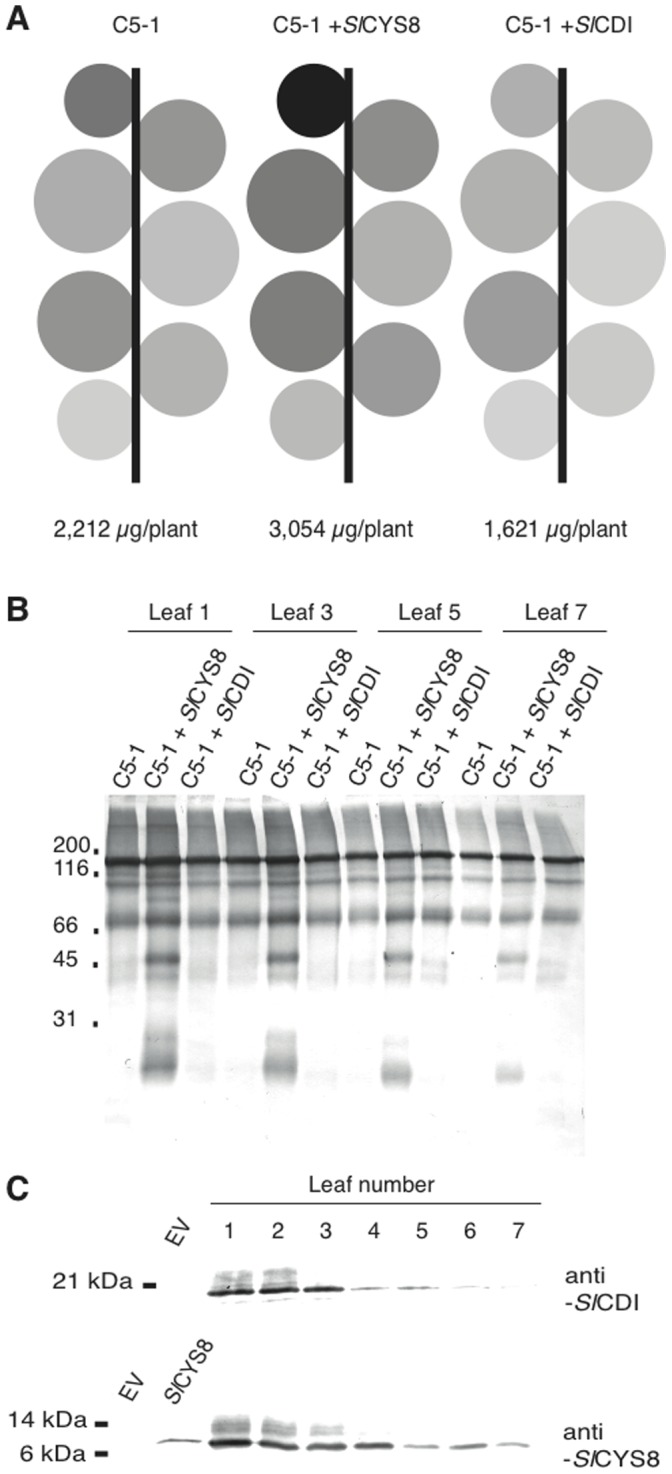
Effect of co-expressed *Sl*CYS8 or *Sl*CDI on C5-1 accumulation. (A) Plants are represented as schematic diagrams where the area of each circle represents the weight of the corresponding leaf. Levels of grey are proportional to the amount of C5-1 on a total soluble protein basis, as determined by quantitative ELISA with a murine IgG standard and normalised to the maximum amount obtained. Leaves were infiltrated with the C5-1 expression vector alone or along with *Sl*CYS8 or *Sl*CDI expression vectors. Each value is the mean of three independent (biological) replicates. Numbers below each plant indicate mean antibody production level for the whole plant. (B) Immunodetection of C5-1 at 6 dpi in Leaf 1, Leaf 3, Leaf 5 and Leaf 7, expressed either alone or along with *Sl*CYS8 or *Sl*CDI. The proteins were detected with alkaline phosphatase-conjugated goat anti-mouse IgG, after SDS-PAGE under non-reducing conditions. (C) Immunodetection of *Sl*CYS8 and *Sl*CDI at 6 dpi in Leaves 1 to 7, following SDS-PAGE under reducing conditions. *Sl*CYS8 was detected with polyclonal primary antibodies raised in rabbit, *Sl*CDI with IgY primary antibodies raised in chicken. The primary antibodies were detected with appropriate alkaline phosphatase-conjugated secondary antibodies. EV, mock infiltration with an empty vector.

An ELISA was performed to quantify C5-1 in whole-leaf protein extracts from tissue harvested at 6 dpi. Antibody levels on a total soluble protein basis generally decreased with leaf age, with the exception of Leaf 5 (Figure 4). Co-expressed *Sl*CYS8 had a positive impact on C5-1 accumulation throughout the plant, whereas *Sl*CDI had a slightly negative impact (Figure 4A). In terms of yield per leaf, *Sl*CYS8 coexpression had a significant positive impact in Leaves 1, 2, 3 and 4, with the effect most marked in Leaf 3 (Table 1). Overall, this effect translated into a 38% increase in the yield of antibody product per plant, from 2.21 to 3.05 mg per plant under our experimental conditions. By comparison, *Sl*CDI had a negative impact on yield in Leaf 1 and Leaf 2, but no significant impact in older leaves. The profile of C5-1 antibody fragments in Leaves 1, 3, 5 and 7 was visualised by Western blotting (Figure 4B). Four major bands were detected in leaves expressing C5-1 alone: a major band at ∼150 kDa, presumably representing the full antibody; and three fragments migrating at about 120, 100 and 80 kDa during SDS-PAGE. Additional bands were detected when *Sl*CYS8 was co-expressed, at about 90, 45 and 25 kDa. The same pattern as C5-1 alone was observed on the immunoblots when *Sl*CDI was co-expressed. Decreased C5-1 product accumulation as a function of leaf age could be observed for all treatments, with a similar decrease in *Sl*CYS8-specific antibody fragments as for the bands found in the control treatment with no co-expressed inhibitor (Figure 4B).

**Table 1:** Antibody product yield (µg) per leaf and per plant in *N. benthamiana* plants transiently expressing C5-1, alone or along with *Sl*CYS8 or *Sl*CDI.

Leaf	C5-1	C5-1+ *Sl*CYS8	C5-1+ *Sl*CDI
1	373±96	535±28*	188±30*
2	440±6	500±41*	276±35*
3	442±21	760±23*	400±48
4	325±76	374±29*	253±53
5	380±113	541±129	354±10
6	204±85	258±99	163±54
7	16±3	25±2	19±8
Total (plant)	2,212±275	3,054±326	1,621±342

* Significantly different to C5-1 expressed alone (ANOVA; *P*<0.05). Values are the mean of three independent (biological) replicates ± SE.

Western blots were performed for *Sl*CYS8 and *Sl*CDI along the leaf-age gradient to assess why co-expressing the inhibitors had more impact in younger leaves, whether positive or negative (Figure 4C). Expression of both inhibitors appeared to decrease with leaf age, with *Sl*CDI and *Sl*CYS8 levels dropping off at Leaf 4 and Leaf 5, respectively. This mirrored the expression profile of C5-1, and suggested that the impact of the inhibitors on C5-1 product accumulation was significant only when a sufficient amount of inhibitor was expressed, irrespective of C5-1 expression level.

### SlCYS8-stabilised C5-1 is Biologically Active

**Figure 5:**
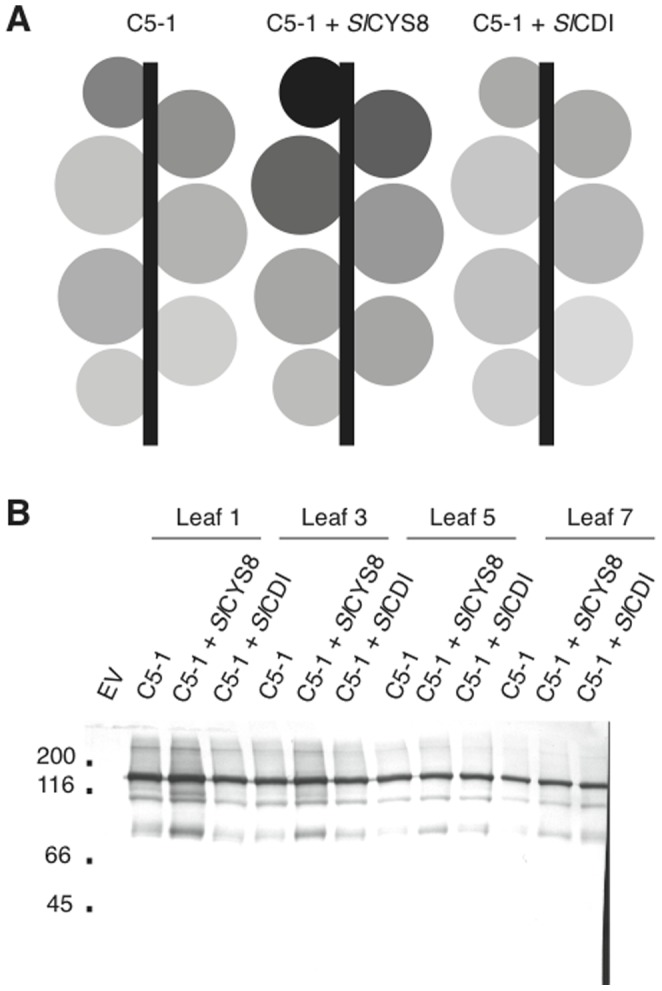
Biological activity of C5-1 expressed alone or along with *Sl*CYS8 or *Sl*CDI. (A) Plants are represented as schematic diagrams, where the area of each circle represents the weight of the corresponding leaf. Levels of grey represent relative C5-1 activities (absorbance units) on a total soluble protein basis, as measured by ELISA and normalised to the maximum amount obtained. Each value is the mean of three biological replicates. (B) Immunodetection of C5-1 activity at 6 dpi in Leaf 1, Leaf 3, Leaf 5 and Leaf 7, with the recombinant antibody expressed alone or along with *Sl*CYS8 or *Sl*CDI. Antibody activity was inferred from binding of human IgG following electroblotting of non-reduced SDS-PAGE-separated proteins onto nitrocellulose sheets. The human IgG antigen of C5-1 was then detected with a goat anti-human conjugated with alkaline phosphatase. EV, mock infiltration with an empty vector.

An ELISA-based C5-1 activity assay was conducted using human IgG antibodies as a test antigen, to determine whether the C5-1 antibody fragments detected at higher levels or newly appeared along with *Sl*CYS8 were biologically active (Figure 5). C5-1 activity levels on a total soluble protein basis generally decreased with leaf age (Figure 5A), reflecting the quantitative profile of C5-1 accumulation (Figure 4). Most interestingly, co-expressed *Sl*CYS8 had a positive impact on C5-1 activity, with a significant effect in the four upper leaves (ANOVA, *P*<0.05) (Figure S1), while *Sl*CDI had generally a negative impact on C5-1 activity (Figure 5A). An activity blot showed which fragments were able to bind antigens, presumably because they contained at least one Fab fragment (Figure 5B). Four bands were detected in leaves expressing C5-1 alone, migrating at ∼150, 120, 100 and 80 kDa and likely corresponding to the four bands detected on qualitative immunoblots (see Figure 4B). The same pattern was observed when *Sl*CYS8 was co-expressed with C5-1, but the bands were more intense. By comparison, the same pattern as C5-1 alone was observed when *Sl*CDI was co-expressed, but with the bands a little less intense. These results demonstrate overall that the additional fragments stabilised in the presence of *Sl*CYS8 (Figure 4), while possibly accounting for some of the increase in C5-1 content, were barely active against human IgG.

### Co-expressed SlCYS8 Extends the Accumulation Phase of C5-1 in Leaves

**Figure 6:**
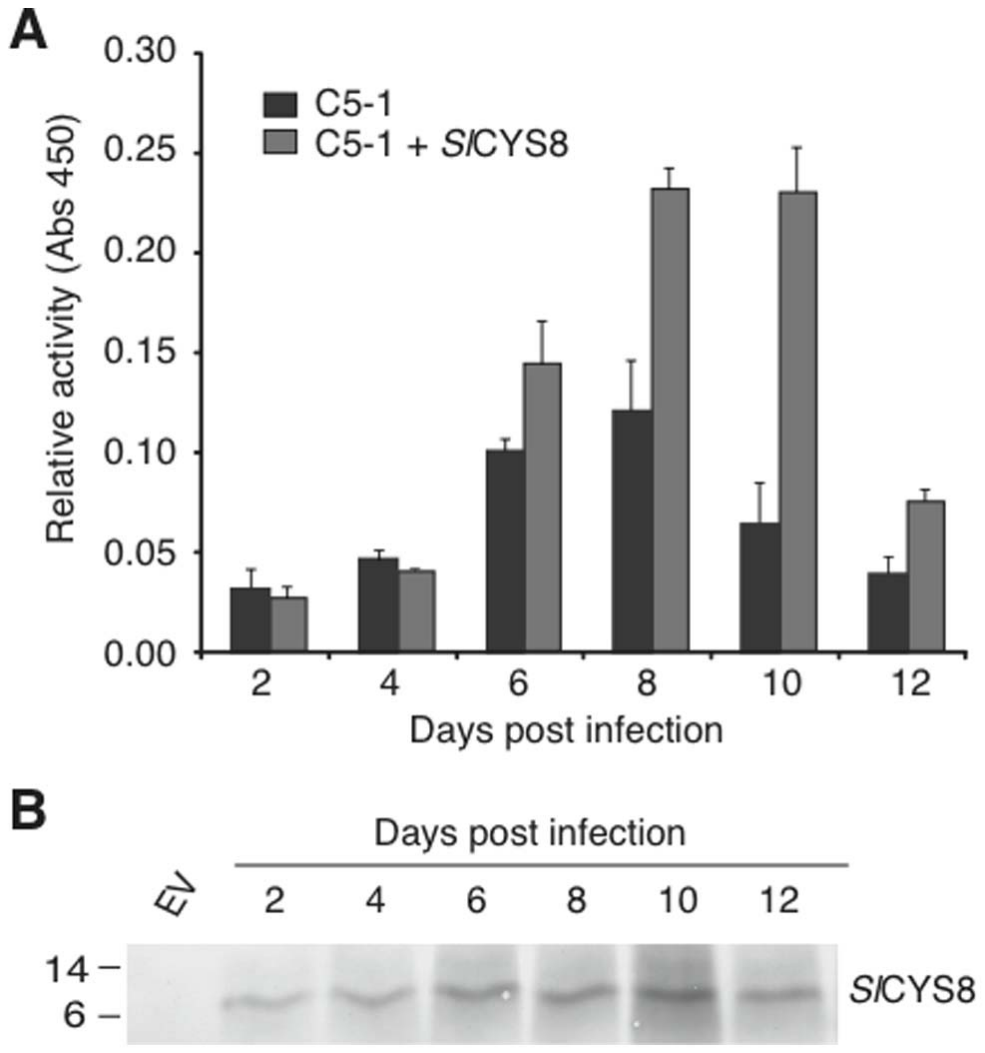
Time-course accumulation of biologically active C5-1 expressed alone or along with *Sl*CYS8. Samples (from Leaf 2, Leaf 3 and Leaf 4) were collected at 2, 4, 6, 8, 10 and 12 dpi from leaf tissue transiently expressing C5-1 alone or along with *Sl*CYS8. (A) The relative activity of C5-1 was measured by ELISA on a total soluble protein basis. Each value is the mean of three independent (biological) replicates ± SE. (B) Immunodetection of *Sl*CYS8 at different time points following SDS-PAGE of leaf proteins under reducing conditions. The cystatin was detected with polyclonal primary antibodies raised in rabbit and an alkaline phosphatase-conjugated goat anti-rabbit secondary antibody. EV, mock infiltration with an empty vector.

Although cathepsin L activity potentially inhibited by *Sl*CYS8 was highest in older infiltrated leaf tissues (Figure 3), *Sl*CYS8 levels in these tissues were apparently too low to have a positive impact on C5-1 accumulation (Figure 4). We reasoned that in the most productive younger leaves, where *Sl*CYS8 had a significant impact, the amount of active C5-1 could be improved as leaf ages if *Sl*CYS8 remained at high enough concentration. Even though leaves are two to three positions further from the apex after the usual 6-days incubation period, *Sl*CYS8 expression was expected to remain high in tissue that is younger at the time of infiltration due to the inherent stability of this protein and to the known effect of P19 to prolong high transgene expression in *N. benthamiana* leaves [41]. Supporting this, sampling tissue every 2 days until day 12 from Leaves 2 to 4 expressing C5-1 alone or along with *Sl*CYS8 showed the impact of *Sl*CYS8 to be much greater at later time points (Figure 6A). There was no apparent impact of *Sl*CYS8 on the accumulation of active C5-1 at 2 and 4 dpi, and a similar positive effect at 6 dpi as measured earlier for 6-days incubations (see Table 1). On the other hand, while the level of C5-1 then increased slightly to reach a maximum at 8 dpi when expressed alone, *Sl*CYS8 had a much greater impact than at earlier time points. Furthermore, whereas the level of C5-1 expressed alone had decreased at 10 dpi, the amount of active antibody was maintained at its highest level in the presence of *Sl*CYS8. Concomitant with the improved levels of active C5-1, *Sl*CYS8 accumulation increased with time post infiltration and reached maximum expression at day 10, after which it decreased (Figure 6B). These results suggest that maintaining high levels of *Sl*CYS8 along the leaf gradient could significantly help to reduce the turnover of C5-1 and allow for a much greater accumulation of active antibody after a slightly longer incubation period.

## Discussion

Transient expression in agroinfiltrated plants is a rapid and simple way of producing large amounts of recombinant proteins in plants. This approach also allows for the parallel modification of host cell processes through the co-expression of accessory proteins permitting, for example, the engineering of recombinant protein post-translational modifications [15], [42] or the implementation of specific protein maturation processes [43]. In the current context of therapeutic protein production where both yield and consistent quality are important for the development of plant-based production platforms [1], [44], there is a need to elucidate metabolic processes governing the production and modification of recombinant proteins *in planta* and to understand how these processes are affected by plant development and physiology. A model was proposed recently to link physiological parameters in *N. tabacum* with predictions for transient expression yields and the costs associated with upstream production and downstream purification [45]. Here we investigated the impact of leaf age and agroinfiltration on major proteolytic activities in leaves of the model protein expression platform *N. benthamiana*, and the possible influence of these activities on recombinant protein yields. By co-expressing full-size IgG such as C5-1 (this study) or the chimeric anti-tumor antibody C2B8 (not shown) with protease inhibitors selected based on their inhibitory potency against the host plant’s proteases, we confirm the potential of controlling endogenous proteolysis to increase the accumulation of functional mammalian antibodies at the whole plant scale, and show that leaf developmental stage has a significant impact on the final outcome. Yield improvements could also be observed using an alternative syringe-free, vacuum-based leaf infiltration procedure (unpublished data), clearly suggesting the general usefulness of protease inhibitors as effective stabilising agents for mammalian antibodies transiently expressed in *N. benthamiana* leaves.

A1 and C1A protease activities monitored in non-infiltrated plants generally increased with leaf age in whole-leaf protein extracts, fitting with the role of proteases in nutrient mobilisation from older tissues as part of a source-sink transition [27]. By contrast, the same activities in apoplast extracts decreased with leaf age, which might reflect a shift away from defence against pathogens, in which proteases are also involved. Agroinfiltration has an extensive impact on the apoplastic proteome of *N. benthamiana* leaves [29], and, accordingly, we found that it had a substantial effect on the distribution of proteolytic activities along the leaf age gradient. Following agroinfiltration, A1 (cathepsin D/E) activity decreased in the apoplast, resulting in no correlation with leaf age. Interestingly, cathepsin D/E activity significantly decreased during C5-1 expression, relative to the control infiltration. The reason for this is unclear but may be related to additional pressures on the secretory system, through which many Asp proteases transit [34]. Cathepsin L activity in the apoplast also decreased following agroinfiltration except in the oldest leaves, resulting in a different profile where C1A-type activity now increased with leaf age for both whole-leaf and apoplast extracts. Both A1 proteases [46], [47] and C1A proteases [48], [49] are known to be upregulated or relocated in response to pathogenic microbes; here we show that this effect may depend on leaf age in *N. benthamiana*.

We transiently expressed the blood-typing murine IgG C5-1 in leaves to assess the impact of variable proteolytic activity profiles on recombinant protein yield along the leaf age gradient. A multi-fragment pattern has been well documented for this antibody whether expressed in transgenic alfalfa [50] or transiently in *N. benthamiana* with viral [51] or non-viral [15] expression systems. While the pattern of immunodetected fragments did not change between leaves, the amount of antibody decreased markedly in older leaves, both in terms of total soluble protein and yield per unit fresh weight. The co-secretion of *Sl*CYS8, an inhibitor of C1A Cys proteases, resulted in the appearance of additional fragments as well as in an increased accumulation of functional antibody all along the leaf age gradient. However, this positive effect of *Sl*CYS8 was statistically significant only in the first four leaves, where *Sl*CYS8 was also expressed well. Together, these observations show that C1A proteases play a role in limiting the accumulation of recombinant antibodies in *N. benthamiana* leaves and that this effect can be significantly limited by the presence of an inhibitor of C1A proteases to increase the yield by nearly 40% on a whole-plant scale.

The non-significant effect of *Sl*CYS8 in older leaves was likely related to low expression of both C5-1 and the recombinant inhibitor on a total soluble protein basis. It has been shown that the CaMV 35S promoter is most efficient in younger leaves of *N. benthamiana* [52], and we found here an increase in cathepsin L activity following agroinfiltration in older leaves (Figure 3). Considering this, the protective effect of *Sl*CYS8 was probably dose-dependent and limited in those leaf tissues exhibiting a high Cys protease *to Sl*CYS8 balance, whether due to a low level of recombinant cystatin or to the age-related increase of endogenous protease activity. In agreement with this hypothesis, continued high-level expression of *Sl*CYS8 over longer incubation times in younger leaves led to an improved protective effect of active C5-1. This result is encouraging as it suggests that further yield improvements could be achieved using expression systems or promoters able to drive high level expression in older tissues. In particular, Leaf 5 and Leaf 6, which account for about 30% of the total biomass (see Figure 2), contributed here to 26% of the antibody yield despite a non-significant effect of *Sl*CYS8 expressed at very low levels.

In summary, the overall yield of a transiently expressed mammalian antibody in *N. benthamiana* leaves appeared to be strongly influenced by endogenous protease activities, which could in turn be mitigated through the co-expression of a suitable recombinant protease inhibitor. We have mapped major endogenous protease activities in relation to leaf age, and shown these activities to be influenced by agroinfiltration and transient protein expression. Based on this, we evaluated the effect of co-expressing protease inhibitors on the resulting yield of a recombinant mammalian antibody, showing in particular the potential of tomato cystatin *Sl*CYS8 to improve the yield of active antibody on a whole plant scale. While this approach for enhancing foreign protein accumulation can result in up to 40% yield improvement per plant, the effect still remains non-significant in oldest leaves where proteolytic activities are stronger, especially in agroinfiltrated tissues. Work is underway to improve the expression of *Sl*CYS8 along the leaf age gradient, with the aim of implementing low Cys protease to *Sl*CYS8 balances in both young, mature and older leaf tissues. Work is also underway to identify inhibitors complementary to *Sl*CYS8, useful in further enhancing the accumulation of antibodies and other proteins. The presence of additional, albeit inactive fragments of C5-1 in *Sl*CYS8-expressing leaves not only confirmed a strong stabilising effect of the tomato cystatin, but also indicated that C5-1 was susceptible to *Sl*CYS8-insensitive proteases *in planta*. Active antibody yields thus could likely be further improved by the co-expression of inhibitors able to prevent antibody fragmentation into *Sl*CYS8-stabilised fragments. No protection effect was observed here for *Sl*CDI, but a significant stabilisation of C5-1 by this inhibitor cannot be excluded under some specific growth conditions [34]. Experiments are currently underway to elucidate this question, keeping in mind the reported impact of plant growth conditions on the degradation and yield of another murine antibody expressed in *N. tabacum* [53].

## Supporting Information

Figure S1 **Activity levels of C5-1 along the leaf age gradient.** Relative C5-1 activities in protein extracts from leaves transiently expressing the antibody alone or along with *Sl*CYS8 or *Sl*CDI were estimated by ELISA, on a total soluble protein basis. Absorbance values for C5-1 alone in each leaf were given an arbitrary value of 1 (dashed line); values with co-expressed *Sl*CYS8 or *Sl*CDI are presented, normalised for each leaf. Each bar is the mean of three independent (biological replicate) values ± SE. Asterisks (*) indicate significant differences compared to C5-1 alone (ANOVA, *P*<0.05).
(PDF)

Table S1 **Proteolytic activities (units/s) in whole-leaf and apoplastic extracts of** ***N. benthamiana*** **leaves either uninfiltrated or six days after infiltration with Agrobacteria containing an empty vector or a C5-1 expression vector.**
(PDF)
