# Supplementary material for: Protection of Recombinant Mammalian Antibodies from Development-Dependent Proteolysis in Leaves of Nicotiana benthamiana
Source: PLoS One. 2013 Jul 23;8(7):e70203. doi: 10.1371/journal.pone.0070203 (PMC3720903; doi:10.1371/journal.pone.0070203)
Supplement: Figure S1 — Activity levels of C5-1 along the leaf age gradient. Relative C5-1 activities in protein extracts from leaves transiently expressing the antibody alone or along with SlCYS8 or SlCDI were estimated by ELISA, on a total soluble protein basis. Absorbance values for C5-1 alone in each leaf were given an arbitrary value of 1 (dashed line); values with co-expressed SlCYS8 or SlCDI are presented, normalised for each leaf. Each bar is the mean of three independent (biological replicate) values ± SE. Asterisks (*) indicate significant differences compared to C5-1 alone (ANOVA, P<0.05). (PDF) [file pone.0070203.s001.pdf]

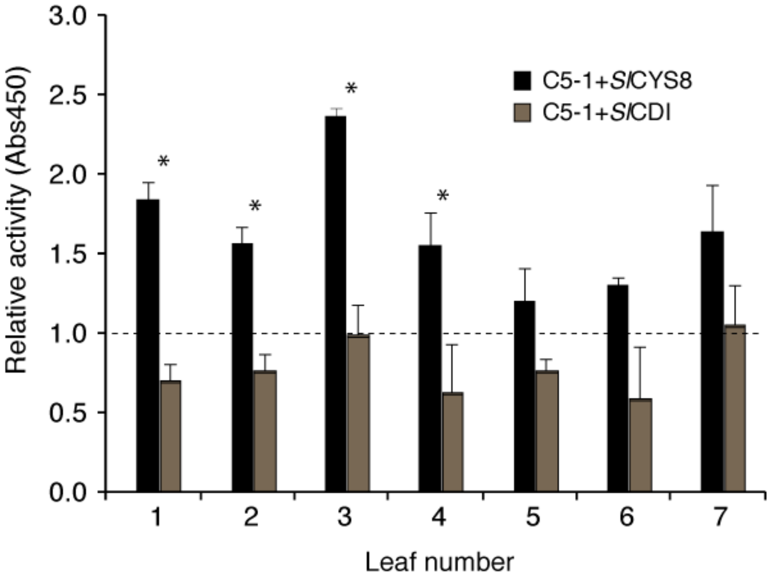

Figure S1. Activity levels of C5-1 along the leaf age gradient. Relative C5-1 activities in protein extracts from leaves transiently expressing the antibody alone or along with *Sl*CYS8 or *Sl*CDI were estimated by ELISA, on a total soluble protein basis. Absorbance values for C5-1 alone in each leaf were given an arbitrary value of 1 (dashed line); values with co-expressed *Sl*CYS8 or *Sl*CDI are presented, normalised for each leaf. Each bar is the mean of three independent (biological replicate) values ± SE. Asterisks (*) indicate significant differences compared to C5-1 alone (ANOVA, $P<0.05$).
