# Supplementary material for: Protection of Recombinant Mammalian Antibodies from Development-Dependent Proteolysis in Leaves of Nicotiana benthamiana
Source: PLoS One. 2013 Jul 23;8(7):e70203. doi: 10.1371/journal.pone.0070203 (PMC3720903; doi:10.1371/journal.pone.0070203)
Supplement: Table S1 — Proteolytic activities (units/s) in whole-leaf and apoplastic extracts of N. benthamiana leaves either uninfiltrated or six days after infiltration with Agrobacteria containing an empty vector or a C5-1 expression vector. (PDF) [file pone.0070203.s002.pdf]

Robert et al. 2013

Table S1 Proteolytic activities (units/s) in whole-leaf and apoplastic extracts of *N. benthamiana* leaves either uninfiltrated or six days after infiltration with Agrobacteria containing an empty vector or a C5-1 expression vector

		Whole leaf extracts			Apoplast extracts		
**Proteases**	**Leaf #**	Uninfiltrated	Empty vector	C5-1 vector	Uninfiltrated	Empty vector	C5-1 vector
Cathepsin L-like	1	18.9 ± 2.7	26.9 ± 4.5	18.6 ± 2.8	82.4 ± 60.5	3.3 ± 0.6	13.3 ± 8.9
	2	28.5 ± 7.2	25.2 ± 1.5	18.9 ± 5.1	29.4 ± 8.3	8.5 ± 4.9	9.8 ± 4.3
	3	47.9 ± 16.7	35.8 ± 5.2	22.8 ± 1.1	44.1 ± 14.9	3.9 ± 0.8	4.2 ± 0.7
	4	56.6 ± 11.9	73.8 ± 7.8	46.9 ± 17.3	32.8 ± 16.5	7.0 ± 1.2	7.3 ± 2.5
	5	89.5 ± 25.8	109.8 ± 34.5	61.1 ± 22.6	25.2 ± 10.9	14.8 ± 3.0	10.3 ± 4.3
	6	98.6 ± 25.9	153.9 ± 23.9	101.9 ± 15.3	36.3 ± 21.3	18.2 ± 3.0	21.6 ± 11.8
	7	153.5 ± 20.9	155.8 ± 49.1	140.5 ± 24.3	12.8 ± 0.0	38.5 ± 13.0	54.4 ± 18.3
Cathepsin D/E-like	1	3.4 ± 1.5	1.3 ± 0.3	2.0 ± 1.0	359.1 ± 43.8	21.9 ± 5.4	6.2 ± 1.5
	2	5.2 ± 2.2	1.2 ± 0.2	2.8 ± 2.0	167.1 ± 63.4	17.8 ± 2.1	7.0 ± 1.9
	3	6.7 ± 3.2	3.7 ± 1.3	0.8 ± 0.1	190.1 ± 99.3	25.1 ± 7.9	10.3 ± 1.1
	4	5.4 ± 1.2	7.9 ± 1.6	1.9 ± 1.0	220.0 ± 51.5	29.2 ± 4.8	15.6 ± 7.5
	5	7.5 ± 2.7	8.3 ± 1.8	1.9 ± 0.4	145.0 ± 30.9	41.9 ± 10.8	15.1 ± 1.7
	6	7.0 ± 1.6	6.1 ± 0.8	1.5 ± 0.7	92.5 ± 37.5	37.9 ± 15.0	36.5 ± 5.2
	7	5.8 ± 0.8	4.2 ± 0.7	3.1 ± 1.4	61.8 ± 0.0	18.7 ± 4.8	37.4 ± 9.7

Values are the mean of three independent (biological replicate) values ± SE.
